# Impact of care management processes and integration of care on blood pressure control in diabetes

**DOI:** 10.1186/1471-2296-14-30

**Published:** 2013-02-27

**Authors:** Ken Wong, Luke Boulanger, Amy Smalarz, Ning Wu, Kimberly Fraser, Jenifer Wogen

**Affiliations:** 1 Novartis Pharmaceuticals Corporation, One Health Plaza, East Hanover, NJ 07936, USA; 2 United Biosource Corporation, 430 Bedford Street Suite 300, Lexington, MA 02420, USA; 3 MedMentis Consulting LLC, 145 Waughaw Road, Towaco, NJ 07082, USA

## Abstract

**Background:**

Fragmentation within health care systems may negatively impact the quality of chronic disease patient care. We sought to evaluate the relationship between care management processes (CMP), integration of services, and blood pressure (BP) control among diabetic patients.

**Methods:**

Retrospective chart reviews were performed for a random sample of adult diabetic hypertensive patients (n = 2,162) from 28 physician organizations in the United States (US). A modified version of the Physician Practice Connection Readiness Survey (PPC-RS) was completed by the chief medical officer at each site. The PPC-RS measured health system organization, delivery system redesign, decision support, clinical information systems, and self-management support, and an integration scale measured structure, functions, and financial risk. Correlations between PPC and integration scores and BP outcomes were assessed using Spearman correlation coefficients.

**Results:**

Approximately 39.9% of diabetic patients had controlled BP. Mean total PPC score across sites was 55, with highest mean scores for health system organization (81), followed by design support (60), clinical information systems (57), self-management support (39), and delivery system redesign (39). Mean integration score was 46 (SD 27, range 4–93), and means of subscores were 64 for structure, 33 for financial risk, and 42 for function. Clinical information systems subscore was correlated with uncontrolled BP (r = −0.38, p < 0.05), while association with total PPC score was strong but not significant at p < 0.05 (r = −0.32). Total integration score and the structure subscore were significantly correlated with BP control (r = 0.38, p < 0.05, and r = 0.49, p < 0.01).

**Conclusions:**

This study suggests that CMP and service integration may be associated with better outcomes in diabetes, though results were mixed and limited by a small number of participating sites. Primary care implementation of integrated electronic medical records may have a beneficial effect on patient outcomes for diabetes and other chronic diseases.

## Background

Fragmentation within health care systems has been hypothesized to be a contributing factor to both suboptimal quality and the high cost associated with the current state of healthcare in the US [1]. The integration of services in physician organizations has been defined as the ability to coordinate functions and activities (inclusive of insurance coverage, payment approaches, and care delivery systems) across separate operating units [2]. This may include vertical integration, such as owning or contracting for physician services, hospital services, urgent care, rehabilitation, and long-term care centers, and/or horizontal integration via the creation of multi-hospital systems [2]. Within specific healthcare practices, structured processes of patient care, in addition to the sum total of all systems involved in the management and delivery of patient healthcare, are referred to as care management processes (CMP) [3]. The use of CMPs among large physician organizations has increased significantly in recent years [4]. The chronic care model (CCM) is a conceptual framework used to organize and characterize these components of comprehensive care for chronic illnesses, which consists of six domains: health system organizations, delivery system redesign, decision support, clinical information systems, self-management support, and community resources and linkages [5,6]. The National Committee for Quality Assurance (NCQA) has developed a survey tool, Physician Practice Connections (PPC), as a basis for evaluating the use of these systems in office practices. A paper version of the PPC, Physician Practice Connection and Readiness Survey (PPC-RS), was adapted to assess the presence of practice systems in the CCM. The PPC-RS is widely used for research purposes, and has been reliability-tested using in-practice audits and has found to be reasonably accurate for research purposes, with a positive predictive value ranging from 55-100% when completed by a group’s medical director [7].

Research conducted by Solberg and colleagues in 2005 used the PPC-RS at 40 practices in Minnesota to demonstrate a relationship between CMPs and diabetic patient outcomes, using a PPC questionnaire targeted at diabetic patient care [8]. While Solberg found a correlation between PPC and most measured outcomes, including glycemic and lipid control, there was no correlation between PPC and BP control. However, there was some effect of CMP on hypertension management in Solberg’s study, as the total PPC-RS score and domain scores for both clinical information systems and decision support were significantly correlated with yearly documented BP measurements (p < 0.05, all comparisons). Since Solberg’s study had limited generalizability based on geographic representation, and a relationship was found between hypertension management (though not BP control) and use of CMP, we sought to evaluate hypertension management among diabetic patients using a more geographically diverse sample of primary care practices. Importantly, Solberg’s 2005 study did not include a measure of service integration, and to our knowledge the relationship between service integration and BP control among diabetic hypertensive patients has not been previously studied. Prior studies have found correlations between the degree of integration of services and PPC-RS scores [9], PPC scores and clinical outcomes in depression [10], and PPC-RS scores and healthcare costs [11].

Conducted prior to the development of the CMP, the Assessment of Chronic Illness Care (ACIC) used a framework similar to the PPC, and early studies using the ACIC demonstrated a relationship between some ACIC subscores and quality of diabetes care [12,13]. A recent cross-sectional analysis among 108 California physician organizations found that greater use of CMPs, as measured via a CMP index based on the CCM by Wagner [5,6], was significantly associated with clinical performance [14]. CMP was related to better diabetes management and improved intermediate outcomes, which incorporated outcomes for coronary artery disease and diabetes, and processes of care (which included clinical measures such as preventive screenings, immunizations, and asthma maintenance). Amundson and colleagues [15] used data reported by the Minnesota Community Measurement (MNCM) to examine clinical outcomes among diabetic patients, and found a significant effect of health insurance product, plan, and physician group on all clinical endpoints evaluated, including glycemic and BP control. Hunt and colleagues [16] evaluated the impact of physician-driven initiatives (including CMPs) for diabetes in Oregon, and demonstrated subsequent improvements in LDL-C and HbA1c testing frequency, increased use of antidiabetic medications, and improved proportions of patients who reached target levels for HbA1C, LDL-cholesterol, and BP. In addition, several studies have used alternate surveys and/or data sources to demonstrate a relationship between quality of care in diabetes and healthcare organizational systems. [15,17]

Fewer studies evaluating the impact of the integration of services have been reported in the literature. Solberg et al. [9] conducted a cross-sectional survey of 97 directors from large medical groups geographically distributed across the US and found that the overall mean PPC-RS score was 58.5% (range = 16-98), with highest scores for health systems. Integration subscores were 53% for function, 30% for structure, and 29% for finance, though a mean overall integration score was not provided. Total PPC-RS score correlated with each integration domain, with the strongest correlation to functional integration. However, this study did not correlate either PPC-RS or integration of services to patient clinical outcomes or quality of care measures.

Since prior research had limited generalizability [8,15,16], but suggested a relationship between hypertension care and CMP [8], the authors sought to further evaluate hypertension management and CMP by using a geographically diverse sample of primary care practices in the US. Furthermore, since prior research did not include a measure of service integration, we sought to evaluate the relationship between diabetic hypertension management and service integration. Thus, the objective of our study was to examine the impact of both care management processes and integration of services on blood pressure control among diabetic hypertensive patients who received care at participating physician organizations.

## Methods

Our cross-sectional study was conducted at 28 physician organizations across the US. A convenience sample of participating sites was identified by field-based outcomes researchers employed by the study sponsor, based on site interest in study participation and size (at least 20 primary care physicians). Participation was independent of the site’s participation in any other research¸ process, or quality improvement initiatives. A random sample of 300 adult hypertensive patients aged 18 years and older was identified by site investigators using random-number generated patient lists. Study subjects were required to have a diagnosis of hypertension (ICD-9-CM 401.x, 402.xx, 403.xx, 404.x, or 405.xx; or written diagnosis of hypertension in doctor’s notes) during the preceding year. Overall diabetes and obesity prevalence were calculated and are presented for all hypertensive patients identified in the original study of all hypertensive patients published previously [18]. For inclusion in this study, patients were also required to have a diagnosis of diabetes during the previous year, which was identified via ICD-9 codes (250.x) or clinical documentation in the patient’s medical record. All study patients thus had co-morbid diagnoses of hypertension and diabetes. Subjects were required to have at least one year history of care with the participating practice, and to have at least 1 office visit during the year preceding the date of data collection. Patients were excluded for pregnancy or participation in a hypertension clinical trial during the preceding year.

Patient demographic and clinical characteristics were assessed via retrospective chart reviews conducted by investigators at each participating site. Data was collected between February 2009 and April 2010; each site collected data for the 1-year period preceding that site’s enrollment date. Collected patient-level data elements included the following: patient age, gender, race/ethnicity, weight, height, smoking status, cardiovascular-related co-morbid conditions (including dyslipidemia and heart failure), two BP measurements (measures from the 2 most recent visits), prescribed antihypertensive medications, and total number of prescribed chronic prescriptions (including hypertension medications). BP control among diabetic patients was defined as systolic BP (SBP) <130 and diastolic BP (DBP) <80 [19,20]. In addition, BP was considered to be uncontrolled if it was higher than goal BP by at least 10 mmHg SBP or 5 mmHg DBP.

**Figure 1:**
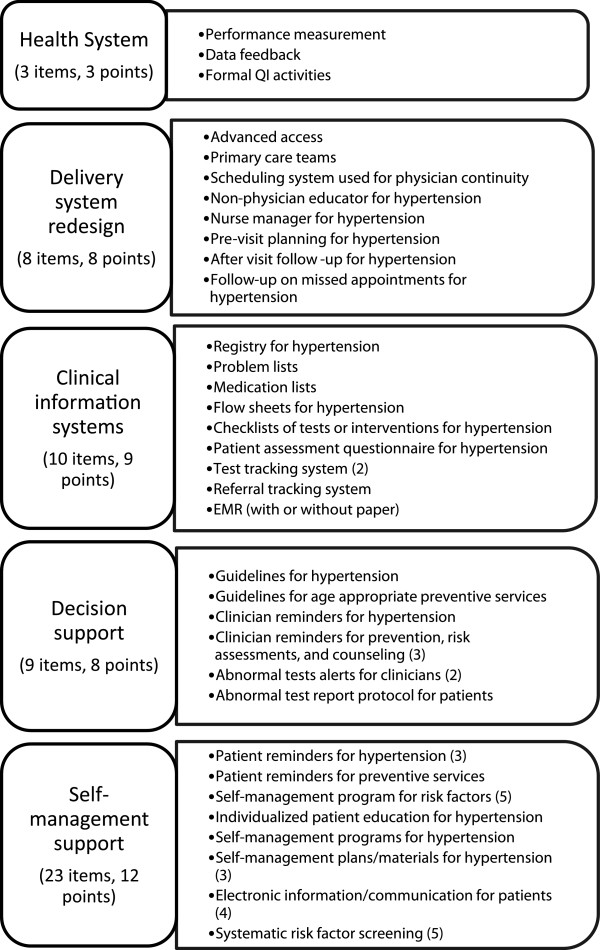
**A modified version of the PPC-RS was administered to the chief medical officer (or equivalent) at each practice site to characterize site-specific care management processes.** The version of the PPC-RS employed in our study measured five out of the six domains in the chronic care model (CCM). Adapted from the NCQA Physician Practice Connection and Readiness Survey (PPC-RS).

**Figure 2:**
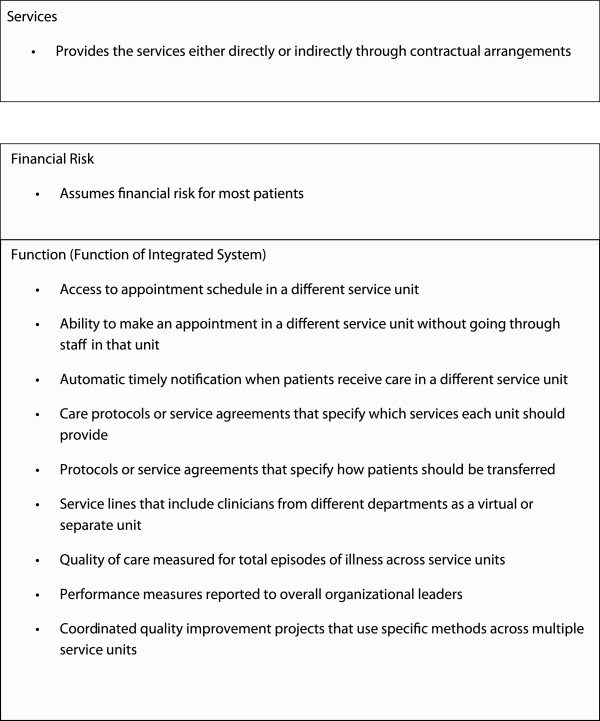
**The PPC-RS included an integration scale that measured structure, functions, and financial risk.** Total integration score was calculated as the mean of these 3 domains, with possible scores of 0 to 100, with a higher score indicating better service integration. Adapted from Solberg et al. 2009 [9].

Site-specific care management processes were assessed using a modified version of the PPC-RS, which was administered to the chief medical officer (or equivalent) at each practice site. The version of the PPC-RS employed in our study measured the following five domains in CCM: health system organization (3 questions), delivery system redesign (8 questions), decision support (9 questions), clinical information systems (10 questions), and self-management support (23 questions) (Figure 1). The PPC-RS was scored by coding each item as present or not present. The score of each domain was calculated as a percentage, using the number of items present as the numerator and the total possible number of items as the denominator. The total PPC score was calculated as the mean of the 5 domain scores, with scores ranging from of 0 to 100, with a higher score indicating more use of CMP. The PPC-RS also included an integration scale that measured structure, functions, and financial risk (Figure 2). Total integration score was calculated as the mean of these 3 domains, with possible scores of 0 to 100 (a higher score indicates better service integration).

This study was approved and monitored by the New England Institutional Review Board (IORG0000444).

### Statistical Analysis

Continuous variables were reported using means and standard deviations, and categorical variables were summarized using frequencies and percentages. PPC score was reported overall and by organizational characteristics. Bivariate correlations between PPC-RS survey scores and aggregated BP outcomes at the organizational level were assessed using Spearman correlation coefficients. The effect of independent variables on BP control was explored by BP control quartiles; aggregated BP outcomes were compared between the lowest (n = 7) and highest (n = 7) quartiles of PPC score. To account for clustering of site-specific variables, generalized linear mixed models (Glimmix) were used to assess the effect of integration score quartile on BP control.

## Results

### Description of sites

Approximately 39% of sites were located in the Midwestern US, while 25% were located in both the Southern and Western regions, and 11% in the Northeast. Most sites (71%) indicated that they had electronic health record (EHR) systems that handle all functions, while 14% indicated that they had EHR systems with separate ordering systems (for lab, radiology, and/or prescriptions). Half of participating sites were physician-owned, while 39% were hospital-owned; 62% had 10 or fewer locations, while 15% had 11–20 locations, and 23% had >20 locations. Approximately 46% of sites employed 50 or more primary care physicians, and 34% had 20–49 primary care physicians. Mean total PPC-RS score across all 28 participating sites was 55% (SD 19) (Table 1). The highest PPC-RS subscore was for health system organization (mean = 81%, SD = 29%), followed by design support (mean = 60%, SD = 29%), clinical information systems (mean = 57%, SD = 16%), delivery system redesign (mean = 39%, SD = 25%), and self-management support (mean = 39%, SD = 26%). The mean overall integration score was 46% (SD = 27%), with mean scores of 64% (SD = 31%) for structure, 42% (SD = 28%) for function, and 33% (SD = 43%) for financial risk.

**Table 1:** Physician practice connections score and integration of services score for all 28 sites

	**Mean**	**Standard deviation**	**Minimum**	**Lower quartile**	**Median**	**Upper quartile**	**Maximum**
Physician Practice Connections Score	55	19	24	38	52	68	94
Health System Organization	81	29	0	67	100	100	100
Delivery System Redesign	39	25	0	25	38	50	100
Clinical Information Systems	57	16	22	44	57	67	89
Design Support	60	29	13	38	56	88	100
Self-Management Support	39	26	8	17	33	63	100
Integration Score	46	27	4	25	48	66	93
Structure	64	31	11	39	61	100	100
Financial Risk	33	43	0	0	0	80	100
Function	42	28	0	20	40	70	80

### Patient characteristics

The entire hypertensive patient population included 8,400 patients, and 2,162 (25.7%) had concomitant diabetes. At the practice level, prevalence of diabetes among hypertensive patients ranged from 11.7%-42.0%. Among the diabetic hypertensive patient population included in this study, mean patient age was 65.4 (SD 12.5) years and 51.2% were female; 39.9% (SD 11.8) had controlled BP, and 30.7% (SD 8.8) had uncontrolled BP. Patient demographic and clinical characteristics are included in Table 2. Both obesity and diabetes prevalence were slightly higher among men as compared to women. While obesity prevalence was similar for Caucasian (44.9%) and African-American (45.1%) patients, the prevalence of diabetes was higher among African-Americans (36.0%) than Caucasians (26.6%), and diabetic African-American patients were more likely to have uncontrolled BP (47.1%) than Caucasians (32.9%). Non-obese patients were more likely to have BP controlled as compared to obese patients (44.7% vs 36.6%). Obesity (38.6%) and diabetes (22.0%) prevalence were lower in the Western US as compared to other geographic regions. Although obesity prevalence was comparable for the Southern, Northeastern, and Midwestern geographic regions, practices located in the Southern US had the highest diabetes prevalence (30.9%). The Southern US also had the lowest proportion of patients with BP controlled (34.2%) and the highest proportion with BP exceeding goal by either >10mmHg SBP or >5mm DBP (38.0%).

**Table 2:** Demographic and clinical factors affecting diabetes and obesity prevalence and BP control among diabetic hypertensive patients from 28 participating practices

	**Mean (SD) obesity prevalence among all study patients (n = 8,400)**	**Mean (SD) DM prevalence among all study patients (n = 8,400)**	**Mean (SD) % with BP control**^**1 **^**among diabetic study patients (n = 2,162)**	**Mean (SD) % with BP uncontrolled**^**2 **^**among diabetic study patients (n = 2,162)**
Gender				
Male	45.5% (8.5%)	27.4% (7.9%)	39.9% (13.9%)	31.8% (10.6%)
Female	43.0% (8.8%)	24.5% (7.6%)	40.1% (12.6%)	29.3% (10.4%)
Age				
<65	53.5% (8.3%)	23.7% (7.7%)	37.5% (14.6%)	31.8% (12.7%)
> = 65	34.4% (8.4%)	28.3% (7.7%)	41.9% (13.3%)	29.9% (9.0%)
Race/ethnicity				
Caucasian	44.9% (7.8%)	26.6% (7.6%)	36.9% (12.8%)	32.9% (7.9%)
African American	45.1% (22.7%)	36.0% (23.2%)	33.5% (25.5%)	47.1% (31.1%)
Hispanic	38.6% (24.5%)	25.4% (28.7%)	42.7% (42.2%)	48.8% (40.2%)
Other/Missing	37.9% (15.9%)	26.1% (19.4%)	42.8% (21.9%)	25.7% (19.1%)
BMI				
<30		18.0% (8.5%)	44.7% (19.2%)	30.9% (15.9%)
> = 30		33.0% (8.4%)	36.6% (12.1%)	32% (9.2%)
Region				
Northeast	45.7% (2.4%)	23.3% (5.2%)	37.7% (13.8%)	26.6% (9.2%)
Midwest	47.5% (8.8%)	25.5% (5.5%)	41.5% (12.6%)	27.9% (7.1%)
South	44.0% (9.2%)	30.9% (8.6%)	34.2% (6.6%)	38.0% (7.7%)
West	38.6% (5.1%)	22.0% (6.7%)	44.1% (13.7%)	29.7% (9.6%)

^1^Controlled BP defined as BP <130/80 mmHg.

^2^Uncontrolled BP defined as SBP > =140 mmHg and/or DBP > =85 mmHg.

**Figure 3:**
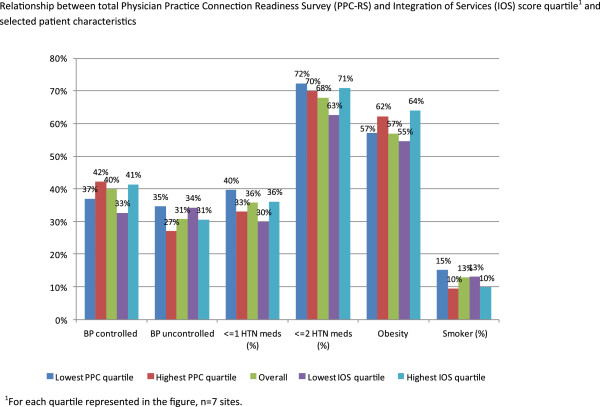
The relationship between total Physician Practice Connection Readiness Survey (PPC-RS) and Integration of Services (IOS) score quartiles and patient BP control, antihypertensive medication regimen, and obesity and smoking status are presented.

Selected patient characteristics, stratified by total PPC score and total integration score quartiles, are presented in Figure 3. Overall, a majority of patients were prescribed 2 or more antihypertensive medications (67.9%). Practices ranked in the highest quartile for both PPC score and total integration score had the lowest smoking prevalence. Mean change in BP between the 2 most recent measurements was −1.3/-0.5 mmHg, and was not related to PPC quartile.

### PPC and integration score results

The relationship between PPC-RS score quartiles and BP control is depicted in Table 3. Sites ranked in the highest quartile of total integration score had better BP control (41.4%) than those in the lowest quartile (32.7%). Compared to the lowest quartile, the 2nd, 3rd, and 4th quartiles had somewhat higher adjusted probability of BP control (p = 0.16, 0.08, and 0.11, respectively). Sites in the highest quartile of total PPC score had somewhat better BP control than those in the lowest quartile (42.2% vs 37.0%, p = NS). This trend towards better BP control was also observed when comparing the 1st and 4th quartiles for the domain scores for health services organization, delivery system redesign, and clinical information systems. A trend towards better control was also observed for the service integration domain scores of structure, function, and, to a lesser extent, financial risk. Correlation values between total PPC score, total integration score, and associated domain scores are also depicted in Table 3. While total PPC score was not significantly correlated with BP control (r = 0.13, p = 0.52), the correlation for total PPC score and uncontrolled BP was strong but not statistically significant (r = −0.32, p = 0.10). A significant correlation was observed for the clinical information systems domain score and uncontrolled BP (r = −0.38, p = 0.04). The correlation value for the overall service integration score and controlled BP was 0.38 (p < 0.05). The integration domain score of structure was significantly correlated with controlled BP (r = 0.49, p < 0.01).

**Table 3:** Relationship between process survey scores, obesity and diabetes prevalence, and BP control among diabetic hypertensive patients

	**N (# practices)**	**BP control among diabetic study patients**		**Correlation of score and BP control (<130/80 mmHg) among DM patients**	**Correlation of score and BP non-control (BP ≥140/85 mmHg) among DM patients**
		**Mean**	**SD**		
Total PPC Score				0.13	−0.32 (p = 0.10)
1st quartile	7	37.0%	10.3%		
4th quartile	7	42.2%	11.3%		
Health System Organization				0.28	−0.18
1st quartile	10	35.9%	9.3%		
2nd quartile	18	42.1%	12.7%		
Delivery System Redesign				0.18	−0.14
1st quartile	12	37.7%	10.8%		
4th quartile	6	41.4%	7.8%		
Clinical Info Systems				0.21	−0.38 (p = 0.04)
1st quartile	9	35.2%	5.8%		
4th quartile	4	46.1%	13.2%		
Design Support				−0.09	−0.30
1st quartile	9	41.3%	9.3%		
4th quartile	4	34.7%	6.8%		
Self-management support				0.04	−0.29
1st quartile	9	36.2%	8.1%		
4th quartile	7	33.8%	8.1%		
Total service integration score				0.38 (p = 0.05)	−0.19
1st quartile	7	32.7%	10.8%		
4th quartile	7	41.4%	8.8%		
Integration- Structure				0.49 (p = 0.01)	−0.30
1st quartile	7	33.8%	10.1%		
4th quartile	14	44.5%	11.4%		
Integration- Financial risk				0.24	−0.15
1st quartile	16	38.0%	13.2%		
4th quartile	6	40.9%	5.9%		
Integration-Function				0.24	−0.06
1st quartile	10	40.5%	12.4%		
4th quartile	4	48.1%	10.0%		

Multivariate analyses using general linear mixed modeling indicated a strong correlation between controlled BP and integration for those practices in the highest integration score quartile (r = 0.34, p = 0.07). The clinical information systems domain score of the PPC-RS was associated with controlled BP, though this also did not reach statistical significance at p < 0.05 (r = 0.36, p = 0.095).

## Discussion

Our cross-sectional study, which employed a modified PPC-RS, found a mean total PPC score of 55% across all sites. However, a considerable amount of variation among practices was observed, as the range of scores was 24%-94%. Highest mean scores were observed for health system organization and design support domain subscores, and lowest subscores were observed for self-management support and delivery system redesign. Overall mean integration score was 46, with highest subscores for structure and lowest for financial risk. Integration of services was more strongly correlated with BP control in our diabetic hypertensive population than the presence of care management processes. A significant correlation between uncontrolled BP and the clinical information systems subscore was observed. A trend was observed between survey scores and BP outcomes, as sites in the highest quartile of total PPC score and total integration score had better BP control than those in the lowest quartile. These findings may not be suitably reflected by statistical comparisons, as our relatively small number of investigative sites resulted in large standard deviations which made formal statistical testing somewhat challenging. These findings are consistent with those observed in a larger hypertensive study cohort (diabetic and non-diabetic patients) using the same 28 study sites that was published previously [18]. That study, however, identified which specific practices were associated with BP control, with results suggesting that the use of physician education regarding patient medication compliance, the use of systematic processes for hypertension screening, and the maintenance of hypertensive patient lists with clinical characteristics were all associated with improved BP control.

BP control was estimated using a cross-sectional retrospective chart review of a sample of diabetic hypertensive patients from practices across the US. We found that 39.9% of our population had BP controlled to <130/80 mmHg, the target measurement recommended by current treatment guidelines [19,20]. Our estimates of BP control in diabetic hypertensive persons is fairly consistent with US normative data, as the most current data from the National Health and Nutrition Examination Survey estimate that across the US, 37.5% of diabetic persons have BP controlled to this level [21]. Variation in diabetes prevalence among investigative sites was evident (11.7%-42.0%); a partial explanation for this wide range in prevalence is geographical and racial/ethnic variation represented by sites. Sites located in the South tended to have more diabetic patients represented in the study, and prevalence was considerably higher among African-Americans.

Previous work to evaluate a potential relationship between care management practices and diabetic patient outcomes has been published by Solberg and colleagues [8] and Amundson et al. [15]. Solberg used the PPC-RS at 40 practices in Minnesota in 2005 to evaluate the relationship between CMP and clinical outcomes in diabetes [8]. Mean total PPC-RS in Solberg’s study was 67.3 (range 32.2-95.8) and thus is somewhat higher than the mean of 55 we observed in the current study; however, the mean PPC-RS for our study’s Midwestern sites was 62.2%, which is similar to the mean PPC-RS observed by Solberg. Among the 40 Minnesota practices studied by Solberg, mean glycemic control (A1C ≤ 8%) was 69% and mean BP control (<130/85 mmHg) was 49%; in our study, mean BP control, defined as <130/80 mmHg was 40%, and it is likely that the discrepancy in BP control definitions between our study and Solberg’s are largely responsible for the disparate BP control estimates. In Solberg’s study, most process and outcome measures were significantly correlated, including glycemic control and LDL-cholesterol. Nevertheless, BP control was not correlated to PPC scores, and a quartile analysis similar to the one conducted in our study did not reveal a trend towards improved BP control and PPC scores. However, in Solberg’s study, the total PPC-RS score and the subscores for clinical information systems and decision support were all significantly correlated with yearly documented BP measurements. Solberg had also used a modified PPC tool to evaluate the relationship between CMPs and outcomes in depression management among Minnesota medical groups. Mean overall PPC scores in this study were 54%, and findings supported a relationship between better quality of care among depressive patients and overall PPC score, as well as for the subscores associated with decision support and delivery system redesign. Our study, by contrast, included 28 primary care practices located throughout the US, and therefore our results may be more generalizeable to the US diabetic hypertensive population that those of Solberg and colleagues. Furthermore, we included an integration of services component to our PPC-RS and found a correlation between BP control and service integration; an integration of services component was not included as part of Solberg’s study.

While our study provides useful information related to service integration and care management process in primary care settings, several caveats are important to consider. Even though our study included data on 2,162 diabetic hypertensive patients at 28 US primary care practices, our study may have benefitted from the inclusion of more physician practices to increase the effective sample size and associated power for statistical comparisons for analysis of BP outcomes and PPC-RS scores. Our study was cross-sectional, and thus the BP measurements recorded were not longitudinal and may not adequately reflect patient BP control over time. Future studies may consider longitudinally following newly-diagnosed patients to more adequately assess patient BP management over time. Site selection was non-random and was based on several factors, including site interest in participation, and the study was not designed to be representative of the demographic composition of the US diabetic hypertensive population as a whole. Thus, it is difficult to ascertain whether selection bias may play a role in some of our study’s findings. We did not collect information on glycemic control, thus we were unable to evaluate this as an endpoint in our diabetic study population. Some additional patient information was not available for analysis, including duration of hypertension or diabetes, patient compliance with prescribed antihypertensive and antidiabetic medication regimen, duration of medication use, and patient insurance status. Furthermore, since the PPC-RS was completed by the chief medical officer at each site, site characteristics may, in a sense, be regarded as self-reported data, and this should be considered in the interpretation of our findings. Nevertheless, despite these limitations, this study provides important information supporting a positive impact of care management processes and service integration in primary care practices’ management of chronic diseases.

## Conclusions

Though our findings were mixed and are limited by a small number of participating practices, our study suggests that the integration of services and the use of care management processes, and clinical information systems in particular, may lead to improved outcomes in diabetic hypertensive persons. Our findings add to the current literature regarding CMP and outcomes in diabetes, as our results may be more generalizeable to primary care across the US diabetic hypertensive population than previously published studies. Furthermore, to our knowledge, ours is the first study to suggest a relationship between service integration and outcomes among diabetic hypertensive persons. Our results suggesting a link between the clinical information systems domain score and improved BP control are particularly important and timely as an increasing number of primary care practices move towards integrated electronic medical records.

## Competing interests

Ken Wong is employed by Novartis Pharmaceuticals Corporation, the study sponsor, and reports stock ownership in Novartis. Jenifer Wogen of MedMentis Consulting received compensation from Novartis and United Biosource Corporation for her work in support of the study and for manuscript preparation. Amy Smalarz, Ning Wu, Kimberly Fraser, and Luke Boulanger have no competing interests to disclose.

## Authors’ contributions

KW participated in study concept and design, analysis and interpretation of data, drafting and critical revision of the manuscript, obtaining funding, and study supervision. JW participated in acquisition of data, analysis and interpretation of data, drafting and critical revision of the manuscript, administrative and technical support, and study supervision. AS, LB, and KF participated in acquisition of data, analysis and interpretation of data, critical revision of the manuscript, provision of study materials, and study supervision. NW participated in analysis and interpretation of data, critical revision of the manuscript, and statistical analysis. All authors read and approved the final manuscript.

## Pre-publication history

The pre-publication history for this paper can be accessed here:

http://www.biomedcentral.com/1471-2296/14/30/prepub
